# Epidemiology of Maternal Nutritional Status and Risk of Adverse Birth Outcomes in Undernourished Mothers with Sickle Cell Disease: A Systematic Review and Meta-Analysis Protocol

**DOI:** 10.3390/mps6050088

**Published:** 2023-09-17

**Authors:** Lauren J. Klein, John Benaiah Ayete-Nyampong, Annette M. Williams, Lori A. Harding, Samuel A. Oppong, Sari Acra, Michael R. DeBaun, Aamer Imdad

**Affiliations:** 1 Department of Pediatrics, D. Brent Polk Division of Pediatric Gastroenterology, Hepatology, and Nutrition at Monroe Carell Jr. Children’s Hospital at Vanderbilt, Nashville, TN 37232, USA; 2 Vanderbilt Institute for Global Health, Vanderbilt University Medical Center, Nashville, TN 37232, USA; m.debaun@vumc.org; 3 Department of Haematology, University of Ghana Medical School, Accra P.O. Box 77, Ghana; johnayete@ymail.com; 4 Center for Knowledge Management, Vanderbilt University Medical Center, Nashville, TN 37232, USA; 5 Department of Obstetrics and Gynaecology, University of Ghana Medical School, Accra P.O. Box 77, Ghana; wak72@yahoo.com; 6 Department of Obstetrics and Gynaecology, Korle-Bu Teaching Hospital, Accra P.O. Box 77, Ghana; 7 Department of Pediatrics, Vanderbilt-Meharry Center of Excellence in Sickle Cell Disease, Vanderbilt University Medical Center, Nashville, TN 37232, USA; 8 Department of Pediatrics, Division of Pediatric Gastroenterology, Hepatology, and Nutrition, University of Iowa Carver College of Medicine, Iowa City, IA 52242, USA

**Keywords:** sickle cell anemia, pregnancy, nutritional status, systematic review

## Abstract

In pregnancies complicated by sickle cell disease (SCD), the maternal-fetal dyad is at high risk for mortality and morbidity. In healthy pregnancies, maternal nutritional status is a critical factor for the healthy growth and development of the fetus. However, there are no reviews of the current research on the nutritional status of pregnant women with SCD and pregnancy outcomes. First, we aim to assess the burden of malnutrition in pregnant women with SCD. Next, we aim to systematically evaluate if pregnant women with SCD who have poor nutritional status are at increased risk for adverse birth outcomes compared to pregnant women with sickle cell disease and normal nutritional status. We will systematically search multiple electronic databases. Our exposure is pregnant women with SCD and poor nutritional status. The primary outcomes of interest include low birth weight (categorical) and birth weight z-scores (continuous). We will also evaluate maternal and perinatal outcomes as secondary outcomes. We will evaluate the risk of bias and overall certainty of evidence with Risk of Bias in Non-randomized Studies—of Interventions (ROBINS-I), and the overall evidence will be assessed using Grading of Recommendation Assessment, Development, and Evaluation (GRADE) criteria. We will pool findings with a meta-analysis if sufficient homogeneity exists among studies. Findings will be published in a peer-reviewed journal and disseminated to SCD advocacy groups. PROSPERO registration number: 429412.

## 1. Introduction

Sickle cell disease is one of the most common inherited genetic diseases worldwide and disproportionately affects low- and middle-income countries, with the greatest burden in sub-Saharan Africa, where 75% of all children with sickle cell disease (SCD) are born annually [1]. Advancements in medical therapies and healthcare access in both low- and high-income countries have greatly increased the survival rates of children with SCD [2,3,4,5,6,7]. Consequently, a substantial portion of women with SCD now reach reproductive age [2,3,4,5,6,7]. However, pregnancy exacerbates the underlying pathophysiology of SCD with higher maternal, fetal, and neonatal mortality and morbidity [1,8,9]. Women with SCD are over twice as likely to have poor fetal and infant growth outcomes compared to women without SCD in both low- and high-income settings [1,8,9].

In pregnancies not complicated by SCD, maternal nutritional status is a key contributor to the healthy growth and development of the fetus during pregnancy [10]. Poor nutritional status in women leads to poor fetal growth, low birth weight, and increased risk of perinatal morbidity and mortality [11,12]. SCD may affect the nutritional status of pregnant women because it causes elevated energy and nutrient requirements [13]. Considering the association between SCD and adverse fetal and infant growth outcomes, as well as the critical role of maternal nutritional status in fetal development unaffected by SCD, there is a need for greater insights into the influence of maternal nutrition in pregnancies complicated by SCD. Therefore, this review aims to evaluate the existing literature concerning the nutritional status of pregnant women with SCD and its implications for maternal-fetal outcomes.

### Objectives

Primary: To determine the burden of malnutrition in pregnant women with SCD. Hypothesis: Malnutrition will be overrepresented in pregnant women with SCD compared to healthy controls.

Secondary: To assess the risk of adverse perinatal outcomes for pregnant women with SCD and poor nutritional status compared to pregnant women with SCD and normal nutritional status. Hypothesis: Pregnant women with SCD and poor nutritional status will have a higher risk of adverse perinatal outcomes compared to pregnant women with SCD and normal nutritional status.

## 2. Experimental Design

### 2.1. Study Type

We will consider observational studies, including both case-control studies and cohort studies with exposed and unexposed groups. We will exclude case reports, case series, commentaries, and review articles. The criteria for inclusion of studies according to the PECO structure [14]—participants, exposure, comparison, and outcome—are listed below. 

### 2.2. Population

We will include studies if participants included pregnant women with SCD, defined as HbSS, HbS beta thalassemia, HbSC, and rare genetic variants (i.e., HbS/D Punjab; HbS/C Harlem; etc.) [2,15]. We will exclude studies detailing maternal sickle cell trait. We will also exclude studies done specifically with participants with other chronic diseases or genetic disorders besides SCD.

### 2.3. Exposure

Our primary exposure of interest is maternal undernutrition in mothers with SCD, which will be defined based on measurements of growth/anthropometry (pre-pregnancy body mass index [BMI], gestational weight gain, weight, height, middle upper arm circumference [MUAC]), and nutritional biomarkers (including but not limited to vitamin D, vitamin A, folate, zinc, and iron). Without associated investigations into the possible nutrition-related cause of anemia, hemoglobin levels will not be considered a nutrition-related biomarker. 

Undernutrition status based on anthropometry will be defined as BMI < 20, MUAC < 23 cm, height and weight z-score < −2, BMI < −2 (wasted) or > 2 standard deviations (overweight and obesity), or gestational weight gain below the Institute of Medicine guidelines [16]. Nutritional biomarkers will be considered suboptimal or low per the definitions of the study authors. We will consider maternal undernutrition if the study population qualifies for undernutrition based on anthropometry or micronutrient status. 

### 2.4. Comparison

For our primary objective, we will compare the prevalence of undernutrition among pregnant women with SCD versus those without SCD. The reason to compare the nutritional status of pregnant mothers with SCD to those without sickle disease is to assess the burden of undernutrition in pregnant mothers with SCD. Our secondary objective for studies with perinatal outcomes is to compare the outcomes of women with SCD with or without poor nutritional status. The reason to compare the birth outcomes in undernourished versus well-nourished mothers with SCD is to assess the association of undernutrition with birth outcomes in pregnant mothers with SCD. 

### 2.5. Outcomes

All outcomes below are dichotomous unless stated otherwise.

#### 2.5.1. Primary Outcomes

- Maternal (primary objective)
  ◦ Maternal undernutrition (defined based on maternal anthropometry and/or micronutrient status)
- Infant (secondary objective)
  ◦ Low birth weight (<2500 g)
  ◦ Very low birth weight (<1500 g)
  ◦ Extremely low birth weight (<1000 g)
  ◦ Birth weight (z-scores) (continuous)

#### 2.5.2. Secondary Outcomes

- Fetal and newborn outcomes (secondary objective)
  ◦ Perinatal mortality (as defined by the study authors)
    ▪ Miscarriage
    ▪ Stillbirth
    ▪ Perinatal mortality
    ▪ Neonatal mortality
  ◦ Morbidity
    ▪ Preterm birth (<37 weeks gestation)
    ▪ Gestational age at birth (continuous)
    ▪ Small for gestational age (as defined by study authors)
  ◦ Anthropometry measured from birth up to 14 days
    ▪ Birth weight (z-scores) (continuous)
    ▪ Birth length (z-scores) (continuous)
    ▪ Birth head circumference (z-scores) (continuous)
- Maternal outcomes (primary and secondary objectives)
  ◦ Mortality
    ▪ Maternal mortality (during pregnancy or within 42 days of pregnancy)
  ◦ Morbidity—from study enrolment until 3 months postpartum (as defined by study authors)
    ▪ Postpartum hemorrhage
    ▪ Cesarean delivery
    ▪ Pre-eclampsia/eclampsia
    ▪ Intensive care unit admissions
    ▪ Acute chest syndrome incidence
    ▪ Vaso-occlusion episodes incidence
    ▪ Stroke incidence

## 3. Procedure

### 3.1. Literature Search

We will conduct systematic searches of the following electronic bibliographic citations databases: EMBASE, PubMed, the Cochrane Central Register of Controlled Trials, Web of Science, CINAHL, WHO Global Index Medicus (including African Index Medicus [AIM] and Latin America and the Caribbean Literature on Health Sciences [LILAC]), BIOSIS, and Google Scholar. We will identify ongoing studies by searching ClinicalTrials.gov. Gray literature and unpublished studies from the preprint services will not be searched. The search will not exclude language. No outcome-related or publication date restrictions will be applied. Additional citations will be gathered by reviewing references of previously published and relevant articles and by using PubMed’s related citations function. We will contact the study authors when further contextual information is necessary to clarify details. Two information specialist librarians on our team will help with the literature searches.

Information specialist librarians carefully crafted expert search statements using a combination of controlled vocabulary and keywords customized for each database to ensure complete search results. Clinical experts within the systematic review team were consulted to refine database strategies. Before conducting the systematic review analysis, all search statements will be re-executed in their respective databases to ensure the capture of the latest evidence. The proposed customized search strategies for the databases are shown in Supplementary Materials Table S1.

### 3.2. Selection of Studies

We will combine searches from all databases in Covidence software and deduplicate the entries. Two independent authors will evaluate the eligibility of studies of inclusion and will document the decisions using Covidence. In the first phase, the authors will review the titles and abstracts to identify studies that may be eligible. In the second phase, the authors will perform a full-text review. Screeners will be blinded to each other’s decisions. Disagreements on study eligibility will be resolved through discussion between screeners and, if needed, adjudicated by a senior author.

We will write the authors to obtain the entire manuscript if a study is only available as an abstract. If we cannot obtain the complete methods and results, we will discuss if there is enough detail in the methods and results of the abstract to include the study in the review. If a study is only available in a non-English language, we will attempt translation utilizing locally available resources. If a study was published in more than one article, then the study will only be counted as one, but we will extract information from all articles as needed. Based on the search strategy and eligibility assessment, we will create a flow diagram following the Preferred Reporting Items for Systematic Reviews and Meta-Analyses (PRISMA) guidelines to visually depict the inclusion and exclusion of studies [17].

### 3.3. Data Extraction

Data will be extracted into a standard data extraction form. Two independent authors will extract data. If there are disagreements between reviewers, these will be resolved by discussion. A third author will also review the manuscript to reach a consensus if required. From each study, the following information will be extracted: study design, study site (country/region), study year, exposure, comparison, outcomes, confounder adjustment, and assessment of the risk of bias.

To decrease bias, we decided a priori a data collection hierarchy when data are presented in multiple formats. When possible, we will extract the most adjusted values, and if adjusted values are not available, we will calculate the odd ratios from the raw data.

### 3.4. Studies with Missing Data

If data are missing for critical variables, we will attempt to contact the study authors. If the study does not report the standard deviation for a continuous outcome, we will attempt to calculate the standard deviation from the available data, including standard error, confidence intervals, and *p*-values. We will contact the study authors if we cannot calculate the standard deviation. If the authors cannot provide the standard deviation, we will utilize a standard deviation from a similar study with a similar population if possible.

### 3.5. Assessment of Risk of Bias in Included Studies

To evaluate the risk of bias, we will use the Risk of Bias in Non-randomized Studies—of Interventions (ROBINS-I). Two authors will independently assign the risk of bias with the ROBINS-I tool [18]. If there is discordance between the risk of bias judgment category for an outcome, the authors will discuss it. The senior author will assign a ROBINS-I score if a disagreement persists.

## 4. Expected Results

We will analyze the randomized and non-randomized studies separately. We will provide a narrative synthesis of all included studies according to the Synthesis Without Meta-analysis (SWiM) guidelines [19]. If more than one study is identified, we will perform meta-analyses. 

We will compare pregnant women classified as having poor nutritional status to those without poor nutritional status within our exposure groups. We will create a composite indicator of poor nutritional status, including all possible exposures reported above.

For outcomes, we will include all outcomes listed in the primary and secondary outcomes listed in the outcomes subsection above. For dichotomous outcomes, we will extract the total number of participants in each group and the number of participants experiencing an event. Dichotomous outcomes will be measured using odds ratios and reported with their corresponding 95% confidence intervals. The measurement of continuous outcomes will involve standardized mean difference effect sizes, which will be reported alongside their corresponding 95% confidence intervals. Meta-analyses will be conducted using the random effects model to address potential heterogeneity resulting from variations in study populations and interventions. We will use Comprehensive Met-Analysis (Biostat, Englewood, NJ, USA) and SPSS 27.0 (IBM, Armonk, NY, USA) software.

We expect that reporting of maternal nutritional status might be in terms of BMI or MUAC. BMI is a commonly used indicator to define malnutrition at the population level; however, it may misclassify some individuals, especially those with increased height or those for whom it is difficult to measure height, such as individuals who are immobile. The MUAC measurement is a better indicator of protein nutrition; however, it is not commonly measured. We aim to include both indicators, BMI and MUAC, to include all the possible studies, and we plan to do a subgroup analysis based on the definition of maternal undernutrition [20].

### 4.1. Assessment of Heterogeneity

Studies brought together for a systematic review vary in the clinical (participants, exposures, outcomes) and the methodological (study design) aspects, which can lead to statistical heterogeneity (variability in the exposure effects) [21]. We will use the χ^2^, I2, and tau statistics to investigate this statistical heterogeneity. The statistical heterogeneity will be considered significant if a *p*-value is < 0.010 or I2 is greater than 50%. We will perform subgroup analysis to investigate statistically significant heterogeneity further. 

### 4.2. Subgroup Analyses

The following a priori subgroup analyses, mainly for quantitative outcomes, are planned:

- Measure of nutritional status:
  ◦ Women with BMI < 20 versus women with normal BMI
  ◦ Women with MUAC < 23 cm versus women with normal MUAC
  ◦ Settings: Low-income country versus middle-income country versus high-income country (If there are limited studies from low-income countries, then low- and middle-income countries will be grouped together.)
- Care delivery model: single-discipline (obstetric) care versus multidisciplinary care (minimum of obstetric and hematology)
- SCD genotype: Sickle cell anemia (homozygous hemoglobin S or hemoglobin Sβ0 thalassemia) versus other SCD genotypes (hemoglobin SC and rare genetic variants)

We will test the difference in subgroups by using the χ^2^ test.

### 4.3. Assessment of Reporting Bias

We will assess small study and publication bias with funnel plots and weighted linear regression (Egger’s) tests for funnel plot asymmetry if the meta-analysis includes at least 10 studies.

### 4.4. Sensitivity Analysis

In order to assess the impact of assumptions made during the analysis on the reliability of the observed outcomes, we will perform the following sensitivity analyses:

- Studies with a high overall risk of bias will be excluded.
- We will include a random versus fixed effect meta-analysis model.

### 4.5. Rating of the Overall Quality of Evidence

The overall quality of evidence will be assessed using the Grading of Recommendations Assessment, Development, and Evaluation (GRADE) approach, which considers factors such as study design, risk of bias (limitations in study design), inconsistency of results, indirectness of evidence, the precision of effect estimates, publication bias, the magnitude of effect, dose-response gradient, and all possible confounding factors (Table 1) [22,23]. We will utilize GRADEpro software for the assessment and to create a summary of findings table [24]. The following quality ratings will be included in the table: very low, low, moderate, and high. Evidence from observational studies starts as low quality, but depending on the above study characteristics, it can be downgraded or upgraded.

### 4.6. Dissemination

We will perform the systematic review and meta-analysis as described herein, and if there are changes or additional analyses from the a priori strategies, this will be detailed in the Methods section of our manuscript.

## Figures and Tables

**Table 1:** Grading of Recommendations Assessment, Development, and Evaluation (GRADE) method to rate the overall quality of evidence. “Adapted from Consultation on the Development of Guidance on How to Incorporate the Results of Modelling into WHO Guidelines. Geneva: World Health Organization; 2017. License: CC BY-NC-SA 3.0 IGO. WHO is not responsible for the content or accuracy of this translation/adaptation.” [23].

Study Design	Quality of Evidence	Lower If	Higher If
Randomized Controlled Trial	High	*Risk of bias* −1 Serious −2 Very serious *Inconsistency* −1 Serious −2 Very serious *Indirectness* −1 Serious −2 Very serious *Imprecision* −1 Serious −2 Very serious *Publication bias* −1 Likely −2 Very Likely	*Large effect* +1 Large +2 Very large *Dose response* +1 Evidence of a gradient *All plausible confounding* +1 Would reduce a demonstrated effect or +1 Would suggest a spurious effect when results show no effect

## Data Availability

We will publish the findings from this review in a peer-reviewed journal. The findings will be disseminated to patient advocacy groups for women with sickle cell disease. All data will be available for review editors and peer reviewers, and raw data will be available for the general public on request.

## Supplementary Materials

The following supporting information can be downloaded at https://www.mdpi.com/article/10.3390/mps6050088/s1: Table S1: Search Strategies.
