# Supplementary material for: Epidemiology of Maternal Nutritional Status and Risk of Adverse Birth Outcomes in Undernourished Mothers with Sickle Cell Disease: A Systematic Review and Meta-Analysis Protocol
Source: Methods Protoc. 2023 Sep 17;6(5):88. doi: 10.3390/mps6050088 (PMC10514847; doi:10.3390/mps6050088)
Supplement: Supplementary file 1 [file mps-06-00088-s001.zip › mps-2470956-supplementary.pdf]

Supplemental Material Table S1. Search Strategies

A. PubMed Search Strategy

Set #	Search
1	(**Anemia, Sickle Cell[mh:noexp]** OR "sickle cell anemia*"[tiab] OR "sickle cell anaemia*"[tiab] OR SCA[tiab] OR "sickle cell disease*"[tiab] OR "sickle cell disorder*"[tiab] OR "sickling disorder*"[tiab] OR sickler[tiab] OR “hemoglobin S disease*"[tiab] OR "haemoglobin S disease*"[tiab] OR "HbS disease*"[tiab] OR **Hemogloblin SC Disease[mh]** OR "hemoglobin SC disease*"[tiab] OR "haemoglobin SC disease"[tiab] OR SCD[tiab] OR HbSC[tiab] OR "Hb SC"[tiab] OR "hemoglobin SS disease*"[tiab] OR "haemoglobin SS disease*"[tiab] OR HbSS[tiab] OR "Hb SS"[tiab] OR "sicle cell"[tiab] OR SCD/SCA[tiab] OR SCA/SCD[tiab] OR "homozygous sickle"[tiab] OR "homozygous SS disease"[tiab] OR "homozygous SS sickle cell"[tiab] OR "sickle cell hemoglobin C disease*"[tiab] OR "sickle cell hemoglobinopath*"[tiab] OR "sickle cell haemoglobinopath*"[tiab] OR "S6/15/C disease*"[tiab] OR "sickle cell hemoglobin C"[tiab] OR "sickle cell haemoglobin C"[tiab] OR “type ss sickle”[tiab] OR “hbs beta”[tiab] OR sickle[tiab] OR sickl[tiab])
2	(**Pregnant Women[mh]** OR **Pregnancy[mh]** OR pregnan*[tiab] OR **Pregnancy Complications[mh**] OR **Pregnancy Trimesters[mh]** OR **Mothers[mh]** OR mother*[tiab] OR **Prenatal Care[mh]** OR **Perinatal Care[mh]** OR **Fetus[mh]** OR fetus*[tiab] OR **Infant, Newborn[mh]** OR newborn*[tiab] OR neonate*[tiab] OR “preterm infant*”[tiab] OR “premature parturition infant*”[tiab] OR “premature infant*”[tiab] OR preemie*[tiab] OR “postparturition”[tiab] OR “post parturition”[tiab] OR **Maternal Nutritional Physiological Phenomena[mh]** OR maternal*[tiab] OR maternity*[tiab] OR childbearing[tiab] OR childbirth[tiab] OR fetomaternal[tiab] OR fetalmaternal[tiab] OR foetalmaternal[tiab] OR postpartum[tiab] OR “post partum”[tiab] OR postpartal[tiab] OR “post partal”[tiab] OR neonatal[tiab] OR prenatal[tiab] OR “pre natal”[tiab] OR perinatal[tiab] OR “peri natal”[tiab] OR antenatal[tiab] OR “ante natal”[tiab] OR fetal[tiab] OR foetal[tiab] OR infant*[tiab] OR infantile[tiab] OR baby[tiab] OR babies[tiab] OR gestation*[tiab] OR stillbirth*[tiab] OR stillborn*[tiab] OR miscarriage*[tiab] OR “spontaneous abortion*”[tiab]OR obstetric*[tiab] OR birth*[tiab] OR born[tiab])
3	(Nutritional[tiab] OR nutrition[tiab] OR **Nutritional Status[mh]** OR **Nutritive Value[mh]** OR **Nutrition Disorders[mh]** OR **Nutrition Assessment[mh]** OR **Diet[mh]** OR diet*[tiab] OR dietary[tiab] OR **Dietary Supplements[mh]** OR **Malnutrition[mh]** OR malnutrition[tiab] OR malnourish*[tiab] OR undernutrition[tiab] OR undernourish*[tiab] OR “placental insufficienc*”[tiab] OR alimentary[tiab] OR food*[tiab] OR **Nutrients[mh]** OR nutrient*[tiab] OR nutritive[tiab] OR macronutrient*[tiab] OR **Micronutrients[mh]** OR micronutrient*[tiab] OR **Vitamins[mh]** OR vitamin*[tiab] OR **Provitamins[mh]** OR provitamin*[tiab] OR **Trace Elements[mh]** OR "trace element*"[tiab] OR "trace mineral*"[tiab] OR **Folic Acid[mh]** OR "folic acid*"[tiab] OR folate[tiab] OR **Ferritins[mh]** OR "ferritin*"[tiab] OR **Copper[mh]** OR copper[tiab] OR **Iron, Dietary[mh]** OR "iron"[tiab] OR **Zinc[mh]** OR zinc[tiab] OR **Magnesium[mh]** OR magnesium[tiab] OR probiotic*[tiab] OR symbiotic*[tiab] OR “ascorbic acid”[tiab] OR “beta carotene”[tiab] OR biotin[tiab] OR carnitine[tiab] OR inositol[tiab] OR niacin[tiab] OR “pantothenic acid”[tiab] OR

	pyridoxine[tiab] OR thiamine[tiab] OR calcitriol[tiab] OR cholecalciferol[tiab] OR ergocalciferols[tiab] OR iodine[tiab] OR manganese[tiab] OR **Recommended Dietary Allowances[mh]** OR "reference daily intake"[tiab] OR "recommended daily intake"[tiab] OR "recommended daily allowance*"[tiab] OR "daily recommended intake"[tiab] OR RDA[tiab] OR **Infant Nutrition Disorders[mh**] OR **Deficiency Diseases[mh]** OR avitaminosis[tiab] OR avitaminoses[tiab] OR “ascorbic acid deficien*”[tiab] OR scurvy[tiab] OR scurvies[tiab] OR hypoascorbemia*[tiab] OR “vitamin C deficien*”[tiab] OR “vitamin A deficien*”[tiab] OR “vitamin B deficien*”[tiab] OR “choline deficien*”[tiab] OR “iodine deficien*”[tiab] OR “protein deficien*”[tiab] OR hyperhomocysteinemia*[tiab] OR homocystinuria*[tiab] OR “cystathionine beta synthase deficien*”[tiab] OR “CBS deficien*”[tiab] OR pellagra*[tiab] OR “riboflavin deficien*”[tiab] OR “thiamine deficien*”[tiab] OR Beriberi[tiab] OR “Wernicke encephalopathy*”[tiab] OR Wernicke*[tiab] OR “Gayet-Wernicke*”[tiab] OR “vitamin B 12 deficien*”[tiab] OR “Addison* anemia*”[tiab] OR “subacute combined degeneration*”[tiab] OR “subacute combined neuropathy degeneration*”[tiab] OR “vitamin B 6 deficien*”[tiab] OR “B6 vitamin deficien*”[tiab] OR “vitamin B6 deficien*”[tiab] OR “pyridoxine deficien*”[tiab] OR **Vitamin D Deficiency[mh]** OR “vitamin D deficien*”[tiab] OR **Vitamin E Deficiency[mh]** OR “vitamin E deficien*”[tiab] OR **Vitamin K Deficiency[mh]** OR “vitamin K deficien*”[tiab] OR **Rickets[mh]** OR rickets[tiab] OR rachitis[tiab] OR rachitides[tiab] OR **Rickets**, **Hypophosphatemic[mh]** OR **Familial Hypophosphatemic Rickets[mh]** OR **Chronic Kidney Disease-Mineral and Bone Disorder[mh]** OR “chronic kidney disease-mineral and bone disorder*”[tiab] OR CKD-MBD[tiab] OR “renal osteodystroph*”[tiab] OR **Osteomalacia[mh]** OR osteomalacia*[tiab] OR **Steatitis[mh]** OR steatitis[tiab] OR **Vitamin K Deficiency Bleeding[mh] OR** “ascorbic acid insufficien*”[tiab] OR “vitamin C insufficien*”[tiab] OR “vitamin A insufficien*”[tiab] OR “vitamin B insufficien*”[tiab] OR “choline insufficien*”[tiab] OR “cystathionine beta synthase insufficien*”[tiab] OR “CBS insufficien*”[tiab] OR “riboflavin insuficien*”[tiab] OR “thiamine insufficien*”[tiab] OR “vitamin B 12 insufficien*”[tiab] OR “vitamin B 6 insufficien*”[tiab] OR “B6 vitamin insufficien*”[tiab] OR “vitamin B6 insufficien*”[tiab] OR “pyridoxine insufficien*”[tiab] OR “vitamin D insufficien*”[tiab] OR “vitamin E insufficien*”[tiab] OR “vitamin K insufficien*”[tiab] OR Biometal*[tiab] OR “vitamin B complex*”[tiab] OR “B vitamin*”[tiab] OR neurobion*[tiab] OR “pteroylpolyglutamic acids*”[tiab] OR pteroylpolyglutamates[tiab] OR tetrahydrofolates[tiab] OR formyltetrahydrofolates[tiab] OR “formyltetrahydrofolic acids”[tiab]OR leucovorin[tiab] OR “folinic acid”[tiab] OR leukovor*[tiab] OR “citrovorum factor”[tiab] OR folinate[tiab] OR 5-Formyltetrahydropteroylglutamate[tiab] OR 5-Formyltetrahydrofolate[tiab] OR Wellcovorin[tiab] OR Levoleucovorin[tiab] OR fusilev[tiab] OR underweight[tiab] OR “under weight”[tiab] OR **“Birth Weight”[mh]** OR “birth weight”[tiab] OR birthweight[tiab] OR **Body weight[mh]** OR “body weight”[tiab] OR **Body Mass Index[mh**] OR “body mass index”[tiab] OR “body mass indice*”[tiab] OR “BMI z-score*”[tiab] OR “small for gestational age”[tiab] OR stunted[tiab] OR stunting[tiab] OR **Starvation[mh]** OR starvation[tiab] OR starved[tiab] OR starving[tiab] OR "fetal growth retardation"[tiab] OR “intrauterine growth restriction”[tiab] OR IUGR[tiab] OR "Intrauterine Growth Retardation"[tiab] OR "fetal growth restriction"[tiab] OR **Bone Density[mh]** OR “bone density”[tiab] OR “muscle wasting”[tiab] OR “wasting syndrome”[tiab] OR “Subjective Global Assessment”[tiab] OR “alpha tocopherol deficiency” OR “e-hypovitaminosis” OR “e-vitaminosis” OR “tocopherol” OR “ascorbate deficien*” OR “C-avitaminosis” OR “C-hypovitaminosis” OR “hypo-vitaminosis” OR “hypovitaminosis” OR “retinol deficien*” OR “keratomalacia” OR “acobalaminosis” OR

	“AdCbl deficien*” OR “adenosylcobalamin deficien*” OR “B12 vitamin*” OR “B 12 vitamin*” OR “Cbl deficien*” OR “Cbl insufficien*” OR “CN-Cbl deficien*” OR “CN-Cbl insufficien*” OR “MeCbl deficien*” OR “MeCbl insufficien*” OR “methylcobalamin deficien*” OR “methylcobalamin insufficien*” OR “calciferol deficien*” OR “calciferol depletion” OR “calciferol insuffien*” OR “colecalciferol deficien*” OR “colecalciferol depletion” OR “colecalciferol insuffien*” OR “D-avitaminosis” OR “menadione deficien*” OR “menadione insufficien*” OR “menadione depletion” OR “menaquinone deficien*” OR “menaquinone insufficien*” OR “menaquinone depletion” OR “phylloquinone deficien*” OR “phylloquinone insufficien*” OR “phylloquinone depletion” OR “cyphytomenadione deficien*” OR “cyphytomenadione insufficien*” OR “cyphytomenadione depletion” OR “corrinoid deficien*” OR “corrinoid insufficien*” OR “corrinoid depletion” OR “multivitamin*” OR “pteroptin” OR “tetrahydrofolic acid derivative” OR “muscle atrophy*” OR “osseous density” OR “bone softening” OR “bone decay” OR “bone dysplagia” OR “pansteatitis” OR “yellow fat disease”)
4	#1 AND #2 AND #3

Abbreviations: * = Truncation symbol; [mh] = Medical Subject Headings (plus all controlled vocabulary terms hierarchically nested below it); [mh: noexp] = Medical Subject Heading only (excluding terms hierarchically nested below the main heading); [pt] = Publication Type; [tiab] = Title and Abstract

B. CINAHL Search Strategy

Set #	Search
1	**(MH "Anemia, Sickle Cell")** OR TI ("sickle cell anemia*" OR "sickle cell anaemia* OR SCA OR "sickle cell disease*" OR "sickle cell disorder*" OR "sickling disorder*" OR sickler OR “hemoglobin S disease*" OR "haemoglobin S disease*" OR "HbS disease*" OR "hemoglobin SC disease*" OR "haemoglobin SC disease" OR SCD OR HbSC OR "Hb SC" OR "hemoglobin SS disease*" OR "haemoglobin SS disease*" OR HbSS OR "Hb SS" OR "sicle cell" OR SCD/SCA OR SCA/SCD OR "homozygous sickle" OR "homozygous SS disease" OR "homozygous SS sickle cell" OR "sickle cell hemoglobin C disease*" OR "sickle cell hemoglobinopath*" OR "sickle cell haemoglobinopath*" OR "SC disease*" OR "sickle cell hemoglobin C" OR "sickle cell haemoglobin C" OR “type ss sickle” OR “hbs beta” OR sickle OR sickl) OR AB ("sickle cell anemia*" OR "sickle cell anaemia* OR SCA OR "sickle cell disease*" OR "sickle cell disorder*" OR "sickling disorder*" OR sickler OR “hemoglobin S disease*" OR "haemoglobin S disease*" OR "HbS disease*" OR "hemoglobin SC disease*" OR "haemoglobin SC disease" OR SCD OR HbSC OR "Hb SC" OR "hemoglobin SS disease*" OR "haemoglobin SS disease*" OR HbSS OR "Hb SS" OR "sicle cell" OR SCD/SCA OR SCA/SCD OR "homozygous sickle" OR "homozygous SS disease" OR "homozygous SS sickle cell" OR "sickle cell hemoglobin C disease*" OR "sickle cell hemoglobinopath*" OR "sickle cell haemoglobinopath*" OR "SC disease*" OR "sickle cell hemoglobin C" OR "sickle cell haemoglobin C" OR “type ss sickle” OR “hbs beta” OR sickle OR sickl)
2	**MH ("Pregnancy+" OR "Pregnancy Complications+" OR "Pregnancy Trimesters+" OR “Mothers+” OR "Prenatal Care" OR "Perinatal Care" OR "Fetus+" OR "Infant, Newborn+" OR "Maternal Nutritional Physiology+" OR “Pregnancy Outcomes” OR “Maternal-Child Care+”)** OR TI (pregnant OR pregnanc* OR mother* OR fetus* OR newborn* OR neonate* OR “preterm infant*” OR “preterm birth*” OR “premature parturition infant*” OR “premature infant*” OR preemie* OR postparturition OR “post parturition” OR “premature birth*” OR maternal* OR maternity* OR childbearing OR childbirth OR fetomaternal OR fetalmaternal OR foetalmaternal OR “birth outcome*” OR postpartum OR “post partum” OR postpartal OR “post partal” OR neonatal OR prenatal OR “pre natal” OR perinatal OR “peri natal” OR antenatal OR “ante natal” OR fetal OR foetal OR infant* OR infantile OR baby OR babies OR gestation* OR stillbirth* OR stillborn* OR miscarriage* OR “spontaneous abortion*”OR obstetric* OR birth* OR born) OR AB (pregnant OR pregnanc* OR mother* OR fetus* OR newborn* OR neonate* OR “preterm infant*” OR “preterm birth*” OR “premature parturition infant*” OR “premature infant*” OR preemie* OR postparturition OR “post parturition” OR “premature birth*” OR maternal* OR maternity* OR childbearing OR childbirth OR fetomaternal OR fetalmaternal OR foetalmaternal OR “birth outcome*” OR postpartum OR “post partum” OR postpartal OR “post partal” OR neonatal OR prenatal OR “pre natal” OR perinatal OR “peri natal” OR antenatal OR “ante natal” OR fetal OR foetal OR infant* OR infantile OR baby OR babies OR gestation* OR stillbirth* OR stillborn* OR miscarriage* OR “spontaneous abortion*”OR obstetric* OR birth* OR born)

3	**MH ("Nutrition+" OR "Nutritional Status" OR "Nutritive Value+" OR "Nutrition Disorders+" OR "Nutritional Assessment" OR "Diet+" OR "Dietary Supplements+" OR "Nutrients+" OR "Vitamins+" OR "Trace Elements+" OR "Minerals+" OR "Ferritin" OR "Copper" OR "Iron" OR "Zinc" OR "Magnesium" OR "Nutritional Requirements+" OR "Deficiency Diseases+" OR "Rickets" OR "Osteomalacia" OR "Body Weight+" OR "Body Mass Index" OR "Fetal Growth Retardation" OR "Bone Density")** OR TI (Nutritional OR nutrition OR diet* OR dietary OR malnutrition OR malnourish* OR undernutrition OR undernourish OR 'placental insufficienc*" OR alimentary OR food* OR "nutrient* OR nutritive OR macronutrients OR micronutrients OR vitamin* OR provitamin* OR "trace element*" OR "folic acid" OR folate OR ferritin OR copper OR iron OR zinc OR magnesium OR probiotic* OR symbiotic* OR "ascorbic acid" OR "beta carotene" OR biotin OR carnitine OR inositol OR niacin OR "pantothenic acid" OR pyridoxine OR thiamine OR calcitriol OR cholecalciferol OR ergocalciferols OR iodine OR manganese OR RDA OR "reference daily intake" OR "recommended daily intake" OR "deficiency disease*" OR avitaminosis OR avitaminoses OR "ascorbic acid deficien*" OR scurvy OR scurvies OR hypoascorbemia* OR "vitamin C deficien*" OR "vitamin A deficien*" OR "vitamin B deficien*" OR "choline deficien*" OR "iodine deficien*" OR "protein deficien*" OR hyperhomocysteinemia* OR homocystinuria* OR "cystathionine beta synthase deficien*" OR "CBS deficien*" OR "pellagra*" OR "riboflavin deficien*" OR "thiamine deficien*" OR Beriberi OR "Wernicke encephalopathy*" OR Wernicke* OR "Gayet-Wernicke*" OR "vitamin B 12 deficien*" OR "Addison* anemia*" OR "subacute combined degeneration*" OR "subacute combined neuropathy degeneration*" OR "vitamin B 6 deficien*" OR "B6 vitamin deficien*" OR "vitamin B6 deficien*" OR "pyridoxine deficien*" OR "vitamin D deficien*" OR "vitamin E deficien*" OR "vitamin K deficien*" OR rickets OR rachitis OR rachitides OR "chronic kidney disease-mineral and bone disorder*" OR "CKD-MBD" OR "renal osteodystroph*" OR osteomalacia* OR steatitis OR "ascorbic acid insufficien*" OR "vitamin C insufficien*" OR "vitamin A insufficien*" OR "vitamin B insufficien*" OR "choline insufficien*" OR "cystathionine beta synthase insufficien*" OR "CBS insufficien*" OR "riboflavin insuficien*"OR "thiamine insufficien*"OR "vitamin B 12 insufficien*" OR "vitamin B 6 insufficien*" OR "B6 vitamin insufficien*" OR "vitamin B6 insufficien*" OR "pyridoxine insufficien*" OR "vitamin D insufficien*" OR "vitamin E insufficien*" OR "vitamin K insufficien*" OR "Biometal*" OR "vitamin B complex*" OR "B vitamin*" OR neurobion*OR "pteroylpolyglutamic acids*" OR pteroylpolyglutamates OR tetrahydrofolates OR formyltetrahydrofolates OR "formyltetrahydrofolic acids" OR leucovorin OR "folinic acid" OR leukovor* OR "citrovorum factor" OR folinate OR 5-Formyltetrahydropteroylglutamate OR 5-Formyltetrahydrofolate OR Wellcovorin OR Levoleucovorin OR fusilev OR underweight OR "under weight" OR "birth weight" OR birthweight OR "body weight" OR "body mass index" OR "body mass indice*" OR "BMI z-score*" OR "small for gestational age" OR stunted OR stunting OR starvation OR starved OR starving OR "fetal growth retardation" OR "intrauterine growth restriction"[tiab] OR IUGR[tiab] OR "Intrauterine Growth Retardation" OR "fetal growth restriction" OR "bone density" OR "muscle wasting" OR "wasting syndrome" OR "Subjective Global Assessment") OR AB (Nutritional OR nutrition OR diet* OR dietary OR malnutrition OR malnourish* OR undernutrition OR undernourish OR 'placental insufficienc*" OR alimentary OR food* OR"nutrient* OR nutritive OR macronutrients OR micronutrients OR vitamin* OR provitamin* OR "trace element*" OR "folic acid" OR folate OR ferritin OR copper OR iron OR zinc OR magnesium OR probiotic* OR symbiotic* OR "ascorbic

	acid” OR “beta carotene” OR biotin OR carnitine OR inositol OR niacin OR “pantothenic acid” OR pyridoxine OR thiamine OR calcitriol OR cholecalciferol OR ergocalciferols OR iodine OR manganese OR RDA OR “reference daily intake” OR “recommended daily intake” OR “deficiency disease*” OR avitaminosis OR avitaminoses OR “ascorbic acid deficien*” OR scurvy OR scurvies OR hypoascorbemia* OR “vitamin C deficien*” OR “vitamin A deficien*” OR “vitamin B deficien*” OR “choline deficien*” OR “iodine deficien*” OR “protein deficien*” OR hyperhomocysteinemia* OR homocystinuria* OR “cystathionine beta synthase deficien*” OR “CBS deficien*” OR “pellagra*” OR “riboflavin deficien*” OR “thiamine deficien*” OR Beriberi OR “Wernicke encephalopathy*” OR Wernicke* OR “Gayet-Wernicke*” OR “vitamin B 12 deficien*” OR “Addison* anemia*” OR “subacute combined degeneration*” OR “subacute combined neuropathy degeneration*” OR “vitamin B 6 deficien*” OR “B6 vitamin deficien*”OR “vitamin B6 deficien*” OR “pyridoxine deficien*” OR “vitamin D deficien*” OR “vitamin E deficien*” OR “vitamin K deficien*” OR rickets OR rachitis OR rachitides OR “chronic kidney disease-mineral and bone disorder*” OR “CKD-MBD” OR “renal osteodystroph*” OR osteomalacia* OR steatitis OR “ascorbic acid insufficien*” OR “vitamin C insufficien*” OR “vitamin A insufficien*” OR “vitamin B insufficien*” OR “choline insufficien*” OR “cystathionine beta synthase insufficien*” OR “CBS insufficien*” OR “riboflavin insuficien*”OR “thiamine insufficien*”OR “vitamin B 12 insufficien*” OR “vitamin B 6 insufficien*” OR “B6 vitamin insufficien*” OR “vitamin B6 insufficien*” OR “pyridoxine insufficien*” OR “vitamin D insufficien*” OR “vitamin E insufficien*” OR “vitamin K insufficien*” OR “Biometal*” OR “vitamin B complex*” OR “B vitamin*” OR neurobion*OR “pteroylpolyglutamic acids*” OR pteroylpolyglutamates OR tetrahydrofolates OR formyltetrahydrofolates OR “formyltetrahydrofolic acids” OR leucovorin OR “folinic acid” OR leukovor* OR “citrovorum factor” OR folinate OR 5-Formyltetrahydropteroylglutamate OR 5-Formyltetrahydrofolate OR Wellcovorin OR Levoleucovorin OR fusilev OR underweight OR “under weight” OR “birth weight” OR birthweight OR “body weight” OR “body mass index*” OR “body mass indice*” OR “BMI z-score*” OR “small for gestational age” OR stunted OR stunting OR starvation OR starved OR starving OR "fetal growth retardation" OR “intrauterine growth restriction*”[tiab] OR IUGR[tiab] OR "Intrauterine Growth Retardation" OR "fetal growth restriction*" OR “bone density” OR “muscle wasting” OR “wasting syndrome*” OR “Subjective Global Assessment” OR “alpha tocopherol deficiency” OR “e-hypovitaminosis” OR “e-vitaminosis” OR “tocopherol” OR “ascorbate deficien*” OR “C-avitaminosis” OR “C-hypovitaminosis” OR “hypo-vitaminosis” OR “hypovitaminosis” OR “retinol deficien*” OR “keratomalacia” OR “acobalaminosis” OR “AdCbl deficien*” OR “adenosylcobalamin deficien*” OR “B12 vitamin*” OR “B 12 vitamin*” OR “Cbl deficien*” OR “Cbl insufficien*” OR “CN-Cbl deficien*” OR “CN-Cbl insufficien*” OR “MeCbl deficien*” OR “MeCbl insufficien*” OR “methylcobalamin deficien*” OR “methylcobalamin insufficien*” OR “calciferol deficien*” OR “calciferol depletion” OR “calciferol insuffien*” OR “colecalciferol deficien*” OR “colecalciferol depletion” OR “colecalciferol insuffien*” OR “D-avitaminosis” OR “menadione deficien*” OR “menadione insufficien*” OR “menadione depletion” OR “menaquinone deficien*” OR “menaquinone insufficien*” OR “menaquinone depletion” OR “phylloquinone deficien*” OR “phylloquinone insufficien*” OR “phylloquinone depletion” OR “cyphytomenadione deficien*” OR “cyphytomenadione insufficien*” OR “cyphytomenadione depletion” OR “corrinoid deficien*” OR “corrinoid insufficien*” OR “corrinoid depletion” OR “multivitamin*” OR “pteroptin” OR “tetrahydrofolic acid derivative” OR “muscle

	atrophy*" OR "osseous density" OR "bone softening" OR "bone decay" OR "bone dysplagia" OR "pansteatitis" OR "yellow fat disease')
4	#1 AND #2 AND #3

Abbreviations: * = Truncation symbol; + = CINAHL Subject Heading plus all the controlled vocabulary terms nested underneath it; MH = CINAHL Controlled Vocabulary Headings (excluding terms hierarchically nested below the main heading); TI = Title; AB = Abstract

C. Embase Search Strategy

Set #	Search
1	(**‘sickle cell anemia’/de** OR **‘hemoglobin SC disease’/de** OR **‘hemoglobin SD disease’/de**) OR (‘sickle cell anemia*’ OR ‘sickle cell anaemia*’ OR SCA OR ‘sickle cell disease*’ OR ‘sickle cell disorder*’ OR ‘sickling disorder*’ OR sickler OR ‘hemoglobin S disease*’ OR ‘haemoglobin S disease*’ OR ‘HbS disease*‘ OR ‘hemoglobin SC disease*‘ OR ‘haemoglobin SC disease*‘ OR SCD OR HbSC OR ‘Hb SC ‘ OR ‘hemoglobin SS disease*‘ OR ‘haemoglobin SS disease*‘ OR HbSS OR ‘Hb SS ‘ OR ‘sicle cell ‘ OR ‘homozygous sickle‘ OR ‘homozygous SS disease‘ OR ‘homozygous SS sickle cell‘ OR ‘sickle cell hemoglobin C disease*‘ OR ‘sickle cell hemoglobinopath*‘ OR ‘sickle cell haemoglobinopath*‘ OR ‘SC disease*‘ OR ‘sickle cell hemoglobin C‘ OR ‘sickle cell haemoglobin C’ OR ‘type ss sickle’ OR ‘bs beta’ OR sickle OR sickl):ab,ti
2	(**‘pregnant woman’/de** OR **‘pregnancy’/exp** OR **‘pregnancy complication’/exp** OR **‘mother’/de** OR **‘prenatal care’/exp** OR **‘perinatal care’/exp** OR **‘fetus’/de** OR **‘infant’/exp** OR **‘birth’/de** OR **‘obstetric patient’/de** OR **‘childbirth’/exp** OR ‘**spontaneous abortion’/exp)** OR (‘pregnan*’ OR ‘fetus*’ OR ‘newborn*’ OR ‘neonate*’ OR ‘preterm infant*’ OR ‘preterm birth*’ OR ‘premature parturition infant*’ OR ‘premature infant*’ OR ‘preemie*’ OR ‘postparturition’ OR ‘post parturition’ OR ‘premature birth*’ OR ‘maternal*’ OR ‘maternity*’ OR ‘childbearing’ OR ‘childbirth’ OR ‘fetomaternal’ OR ‘fetalmaternal’ OR ‘foetalmaternal’ OR ‘birth outcome*’ OR ‘postpartum’ OR ‘post partum’ OR ‘postpartal’ OR ‘post partal’ OR ‘neonatal’ OR ‘prenatal’ OR ‘pre natal’ OR ‘perinatal’ OR ‘peri natal’ OR ‘antenatal’ OR ‘ante natal’ OR ‘fetal’ OR ‘foetal’ OR ‘infant*’ OR ‘infantile’ OR ‘baby’ OR ‘babies’ OR ‘gestation*’ OR ‘stillbirth*’ OR ‘stillborn*’ OR ‘miscarriage*’ OR ‘spontaneous abortion*’ OR ‘obstetric*’ OR ‘birth*’ OR ‘born’):ab,ti
3	(**‘nutrition’/exp** OR **‘nutritional disorder’/exp** OR **‘placental insufficiency’/de** OR **‘vitamin’/exp** OR **‘trace element’/exp** OR **‘ferritin’/de** OR **‘copper’/de** OR **‘probiotic agent’/exp** OR **‘ascorbic acid’/exp** OR **‘inositol’/de** OR **‘nicotinic acid’/de** OR **‘iodine’/de** OR **‘manganese’/de** OR **‘hyperhomocysteinemia’/de** OR **‘homocystinuria’/de** OR **‘Wernicke encephalopathy’/de** OR ‘**Addison disease’/de** OR **‘subacute combined degeneration’/de** OR **‘rickets’/exp** OR **‘chronic kidney disease-mineral and bone disorder’/exp** OR **‘osteodystrophy’/de** OR **‘osteomalacia’/exp** OR **‘steatitis’/de** OR **‘body weight’/exp** OR **‘body mass’/de** OR **‘growth disorder’/exp** OR **‘bone density’/exp** OR **‘muscle atrophy’/de** OR **‘bone dysplasia’/exp**) OR (‘nutrition*’ OR ‘nutritive’ OR ‘diet’ OR ‘malnutrition’ OR ‘malnourish*’ OR ‘undernutrition’ OR ‘placent*’ OR ‘alimentary’ OR ‘food’ OR ‘nutrient*’ OR ‘nutritive’ OR ‘macronutrient*’ OR ‘micronutrient*’ OR ‘vitamin*’ OR ‘provitamin*’ OR ‘trace element*’ OR ‘trace mineral*’ OR ‘folic acid’ OR ‘folate*’ OR ‘ferritin*’ OR ‘copper’ OR ‘iron’ OR ‘zinc’ OR ‘magnesium’ OR ‘probiotic’ OR ‘ascorbic acid’ OR ‘beta carotene’ OR ‘biotin’ OR ‘carnitine’ OR ‘inositol’ OR ‘niacin’ OR ‘pathothenic acid’ OR ‘pyridoxine’ OR ‘thiamine’ OR ‘calcitriol’ OR ‘cholecalciferol’ OR ‘ergocalciferol*’ OR ‘nicotinic acid*’ OR ‘iodine’ OR ‘manganese’ OR ‘reference daily intake’ OR ‘recommended daily intake’ OR ‘recommended daily allowance*’ OR ‘daily recommended intake’ OR ‘RDA’ OR ‘hyperhomocysteinemia’ OR ‘homocystinuria’ OR ‘Wernicke’ OR ‘Addison dis’ OR ‘Addison an*emia*’ OR ‘subacute combined degeneration’ OR ‘subacute combined

	neuropathy degeneration*' OR 'rickets' OR 'deficiency dis*' OR 'avitaminosis' OR 'scurvy' OR 'scurvies' OR 'hypoascorbemia*' OR 'vitamin C' OR 'vitamin A' OR 'vitamin B' OR 'choline' OR 'protein' OR 'cystathionine beta synthase deficien*' OR 'CBS deficien*' OR 'pellagra' OR 'riboflavin' OR 'beriberi' OR 'B 6 vitamin*' OR 'chronic kidney disease mineral and bone disease' OR 'renal osteodystroph*' OR 'Osteomalacia' OR 'steatitis' OR 'biometal' OR 'neurobion' OR 'pteroylpolyglutamic acids*' OR 'pteroylpolyglutamate*' OR 'tetrahydrofolate*' OR 'formyltetrahydrofolate*' OR 'folinic acid' OR 'formyltetrahydrofolic' OR 'leucovorin' OR 'leukovor*' OR 'citrovorum factor' OR 'folinate' OR '5-Formyltetrahydropteroylglutamate' OR '5-Formyltetrahydrofolate' OR 'wellcovorin' OR 'Levoleucovorin' OR 'fusilev' OR 'underweight' OR 'under weight' OR 'birthweight' OR 'body weight' OR 'body mass' OR 'BMI z-score' OR 'growth disorder' OR 'stunted' OR 'stunting' OR 'starvation' OR 'starve*' OR 'starving' OR 'growth retardation' OR 'intrauterine growth restriction' OR 'IGR' OR 'fetal growth restriction' OR 'bone density' OR 'muscle wasting' OR 'alpha tocopherol deficiency' OR 'e-hypovitaminosis' OR 'e-vitaminosis' OR 'tocopherol' OR 'ascorbate deficien*' OR 'C-avitaminosis' OR 'C-hypovitaminosis' OR 'hypo-vitaminosis' OR 'hypovitaminosis' OR 'retinol deficien*' OR 'keratomalacia' OR 'acobalaminosis' OR 'AdCbl deficien*' OR 'adenosylcobalamin deficien*' OR 'B12 vitamin*' OR 'B 12 vitamin*' OR 'Cbl deficien*' OR 'Cbl insufficien*' OR 'CN-Cbl deficien*' OR 'CN-Cbl insufficien*' OR 'MeCbl deficien*' OR 'MeCbl insufficien*' OR 'methylcobalamin deficien*' OR 'methylcobalamin insufficien*' OR 'calciferol deficien*' OR 'calciferol depletion' OR 'calciferol insuffien*' OR 'colecalciferol deficien*' OR 'colecalciferol depletion' OR 'colecalciferol insuffien*' OR 'D-avitaminosis' OR 'menadione deficien*' OR 'menadione insufficien*' OR 'menadione depletion' OR 'menaquinone deficien*' OR 'menaquinone insufficien*' OR 'menaquinone depletion' OR 'phylloquinone deficien*' OR 'phylloquinone insufficien*' OR 'phylloquinone depletion' OR 'cyphytomenadione deficien*' OR 'cyphytomenadione insufficien*' OR 'cyphytomenadione depletion' OR 'corrinoid deficien*' OR 'corrinoid insufficien*' OR 'corrinoid depletion' OR 'multivitamin*' OR 'pteroptin' OR 'tetrahydrofolic acid derivative' OR 'muscle atrophy*' OR 'osseous density' OR 'bone softening' OR 'bone decay' OR 'bone dysplagia' OR 'pansteatitis' OR 'yellow fat disease'):ab,ti OR (vitamin* NEXT/4 depletion):ab,ti
4	#1 AND #2 AND #3

Abbreviations: * = Truncation symbol; :ab,ti = Abstract and Title; /de = Embase Medical Subject Headings (excluding terms hierarchically nested below the main heading); /exp = Exploded Embase Medical Subject Heading (including terms hierarchically nested below the main heading)

D. Web of Science Search Strategy

Set #	Search
1	TS= ("sickle cell anemia*" OR "sickle cell anaemia*" OR "sickle cell dis*" OR "sickling dis*" OR sickler OR “h*emoglobin S dis*” OR “HbS dis* OR “h*emoglobin SC dis*” OR SCD OR HbSC OR “Hb SC” OR “h*emoglobin SS dis*” OR HbSS OR “Hb SS” OR “sicle cell” OR SCD/SCA OR SCA/SCD OR “homozygous sickle” OR “homozygous SS dis*” OR “homozygous SS sickle cell" OR "sickle cell h*emoglobin C" OR "sickle cell h*emoglobinopath*" OR "SC dis* OR “type ss sickle” OR “hbs beta” OR sickle OR sickl)
2	TS=(pregnan* OR mother* OR fetus* OR newborn* OR infant* OR neonat* OR “premature parturition” OR preemie* OR postparturition OR “post parturition” OR birth* OR maternal* OR maternity OR childbearing OR childbirth OR fetomaternal OR fetalmaternal OR foetalmaternal OR postpartum OR “post partum” OR postpartal OR “post partal” OR prenatal OR “pre natal” OR perinatal OR “peri natal” OR antenatal OR “ante natal” OR fetal OR foetal OR baby OR babies OR gestation* OR stillbirth* OR stillborn* OR miscarriage* OR “spontaneous abortion*” OR obstetric* OR born)
3	TS=(nutrition* OR diet* OR dietary OR malnutrition OR malnourish* OR undernutrition OR undernourish* OR “placental insufficienc*” OR alimentary OR food* OR nutrient* OR nutritive OR macronutrient* OR micronutrient* OR vitamin* OR provitamin* OR "trace element*" OR "trace mineral*" OR “folic acid*" OR folate OR ferritin* OR copper OR iron OR zinc OR magnesium OR probiotic* OR symbiotic* OR “ascorbic acid” OR “beta carotene” OR biotin OR carnitine OR inositol OR niacin OR “pantothenic acid” OR pyridoxine OR thiamine OR calcitriol OR cholecalciferol OR ergocalciferols OR iodine OR manganese OR “recommended dietary allowance*” OR "reference daily intake" OR "recommended daily intake" OR "recommended daily allowance*" OR "daily recommended intake" OR RDA OR “infant nutrition disorder*” OR “deficiency disease*” OR avitaminosis OR avitaminoses OR scurvy OR scurvies OR hypoascorbemia* OR “vitamin C deficien*” OR “vitamin A deficien*” OR “vitamin B deficien*” OR “choline deficien*” OR “iodine deficien*” OR “protein deficien*” OR hyperhomocysteinemia* OR homocystinuria* OR “cystathionine beta synthase deficien*” OR “CBS deficien*” OR pellagra* OR “riboflavin deficien*” OR “thiamine deficien*” OR Beriberi OR “Wernicke encephalopathy*” OR Wernicke* OR “Gayet-Wernicke*” OR “vitamin B 12 deficien*” OR “Addison* anemia*” OR “subacute combined degeneration*” OR “subacute combined neuropathy degeneration*” OR “vitamin B 6 deficien*” OR “B6 vitamin deficien*” OR “vitamin B6 deficien*” OR “pyridoxine deficien*” OR “vitamin D deficien*” OR “vitamin E deficien*” OR “vitamin K deficien*” OR rickets OR rachitis OR rachitides OR “chronic kidney disease-mineral and bone disorder*” OR CKD-MBD OR “renal osteodystroph*” OR osteomalacia* OR steatitis OR “ascorbic acid insufficien*” OR “vitamin C insufficien*” OR “vitamin A insufficien*” OR “vitamin B insufficien*” OR “choline insufficien*” OR “cystathionine beta synthase insufficien*” OR “CBS insufficien*” OR “riboflavin insuficien*” OR “thiamine insufficien*” OR “vitamin B 12 insufficien*” OR “vitamin B 6 insufficien*” OR “B6 vitamin insufficien*” OR “vitamin B6 insufficien*” OR “pyridoxine insufficien*” OR “vitamin D insufficien*” OR “vitamin E insufficien*” OR “vitamin K insufficien*” OR Biometal* OR “vitamin B complex*” OR “B vitamin*” OR neurobion* OR “pteroylpolyglutamic acids*” OR pteroylpolyglutamates OR tetrahydrofolates OR formyltetrahydrofolates OR “formyltetrahydrofolic acids” OR leucovorin OR “folinic acid” OR leukovor* OR “citrovorum

	factor” OR folinate OR 5-Formyltetrahydropteroylglutamate OR 5-Formyltetrahydrofolate OR Wellcovorin OR Levoleucovorin OR fusilev OR underweight OR “under weight” OR “birth weight” OR birthweight OR “body weight” OR “body mass index” OR “body mass indice*” OR “BMI z-score*” OR “small for gestational age” OR stunted OR stunting OR starvation OR starved OR starving OR "fetal growth retardation" OR “intrauterine growth restriction” OR IUGR OR "intrauterine growth retardation" OR "fetal growth restriction" OR “bone density” OR “muscle wasting” OR “wasting syndrome” OR “Subjective Global Assessment” OR “alpha tocopherol deficiency” OR “e-hypovitaminosis” OR “e-vitaminosis” OR tocopherol OR “ascorbate deficien*” OR “C-avitaminosis” OR “C-hypovitaminosis” OR “hypo-vitaminosis” OR hypovitaminosis OR “retinol deficien*” OR keratomalacia OR acobalaminosis OR “AdCbl deficien*” OR “adenosylcobalamin deficien*” OR “B12 vitamin*” OR “B 12 vitamin*” OR “Cbl deficien*” OR “Cbl insufficien*” OR “CN-Cbl deficien*” OR “CN-Cbl insufficien*” OR “MeCbl deficien*” OR “MeCbl insufficien*” OR “methylcobalamin deficien*” OR “methylcobalamin insufficien*” OR “calciferol deficien*” OR “calciferol depletion” OR “calciferol insuffien*” OR “colecalciferol deficien*” OR “colecalciferol depletion” OR “colecalciferol insuffien*” OR “D-avitaminosis” OR “menadione deficien*” OR “menadione insufficien*” OR “menadione depletion” OR “menaquinone deficien*” OR “menaquinone insufficien*” OR “menaquinone depletion” OR “phylloquinone deficien*” OR “phylloquinone insufficien*” OR “phylloquinone depletion” OR “cyphytomenadione deficien*” OR “cyphytomenadione insufficien*” OR “cyphytomenadione depletion” OR “corrinoid deficien*” OR “corrinoid insufficien*” OR “corrinoid depletion” OR multivitamin* OR pteroptin OR “tetrahydrofolic acid derivative” OR “muscle atrophy*” OR “osseous density” OR “bone softening” OR “bone decay” OR “bone dysplagia” OR pansteatitis OR “yellow fat disease”)
4	#1 AND #2 AND #3

Abbreviations: *= Truncation and character wildcard symbol; TS= keyword search

E. Biosis Search Strategy

Set #	Search
1	TS= ("sickle cell anemia*" OR "sickle cell anaemia*" OR "sickle cell dis*" OR "sickling dis*" OR sickler OR “h*emoglobin S dis*” OR “HbS dis* OR “h*emoglobin SC dis*” OR SCD OR HbSC OR “Hb SC” OR “h*emoglobin SS dis*” OR HbSS OR “Hb SS” OR “sicle cell” OR SCD/SCA OR SCA/SCD OR “homozygous sickle” OR “homozygous SS dis*” OR “homozygous SS sickle cell" OR "sickle cell h*emoglobin C" OR "sickle cell h*emoglobinopath*" OR "SC dis* OR “type ss sickle” OR “hbs beta” OR sickle OR sickl)
2	TS=(pregnan* OR mother* OR fetus* OR newborn* OR infant* OR neonat* OR “premature parturition” OR preemie* OR postparturition OR “post parturition” OR birth* OR maternal* OR maternity OR childbearing OR childbirth OR fetomaternal OR fetalmaternal OR foetalmaternal OR postpartum OR “post partum” OR postpartal OR “post partal” OR prenatal OR “pre natal” OR perinatal OR “peri natal” OR antenatal OR “ante natal” OR fetal OR foetal OR baby OR babies OR gestation* OR stillbirth* OR stillborn* OR miscarriage* OR “spontaneous abortion*” OR obstetric* OR born)
3	TS=(nutrition* OR diet* OR dietary OR malnutrition OR malnourish* OR undernutrition OR undernourish* OR “placental insufficienc*” OR alimentary OR food* OR nutrient* OR nutritive OR macronutrient* OR micronutrient* OR vitamin* OR provitamin* OR "trace element*" OR "trace mineral*" OR “folic acid*" OR folate OR ferritin* OR copper OR iron OR zinc OR magnesium OR probiotic* OR symbiotic* OR “ascorbic acid” OR “beta carotene” OR biotin OR carnitine OR inositol OR niacin OR “pantothenic acid” OR pyridoxine OR thiamine OR calcitriol OR cholecalciferol OR ergocalciferols OR iodine OR manganese OR “recommended dietary allowance*” OR "reference daily intake" OR "recommended daily intake" OR "recommended daily allowance*" OR "daily recommended intake" OR RDA OR “infant nutrition disorder*” OR “deficiency disease*” OR avitaminosis OR avitaminoses OR scurvy OR scurvies OR hypoascorbemia* OR “vitamin C deficien*” OR “vitamin A deficien*” OR “vitamin B deficien*” OR “choline deficien*” OR “iodine deficien*” OR “protein deficien*” OR hyperhomocysteinemia* OR homocystinuria* OR “cystathionine beta synthase deficien*” OR “CBS deficien*” OR pellagra* OR “riboflavin deficien*” OR “thiamine deficien*” OR Beriberi OR “Wernicke encephalopathy*” OR Wernicke* OR “Gayet-Wernicke*” OR “vitamin B 12 deficien*” OR “Addison* anemia*” OR “subacute combined degeneration*” OR “subacute combined neuropathy degeneration*” OR “vitamin B 6 deficien*” OR “B6 vitamin deficien*” OR “vitamin B6 deficien*” OR “pyridoxine deficien*” OR “vitamin D deficien*” OR “vitamin E deficien*” OR “vitamin K deficien*” OR rickets OR rachitis OR rachitides OR “chronic kidney disease-mineral and bone disorder*” OR CKD-MBD OR “renal osteodystroph*” OR osteomalacia* OR steatitis OR “ascorbic acid insufficien*” OR “vitamin C insufficien*” OR “vitamin A insufficien*” OR “vitamin B insufficien*” OR “choline insufficien*” OR “cystathionine beta synthase insufficien*” OR “CBS insufficien*” OR “riboflavin insuficien*” OR “thiamine insufficien*” OR “vitamin B 12 insufficien*” OR “vitamin B 6 insufficien*” OR “B6 vitamin insufficien*” OR “vitamin B6 insufficien*” OR “pyridoxine insufficien*” OR “vitamin D insufficien*” OR “vitamin E insufficien*” OR “vitamin K insufficien*” OR Biometal* OR “vitamin B complex*”

	OR “B vitamin*” OR neurobion* OR “pteroylpolyglutamic acids*” OR pteroylpolyglutamates OR tetrahydrofolates OR formyltetrahydrofolates OR “formyltetrahydrofolic acids” OR leucovorin OR “folinic acid” OR leukovor* OR “citrovorum factor” OR folinate OR 5-Formyltetrahydropteroylglutamate OR 5-Formyltetrahydrofolate OR Wellcovorin OR Levoleucovorin OR fusilev OR underweight OR “under weight” OR “birth weight” OR birthweight OR “body weight” OR “body mass index” OR “body mass indice*” OR “BMI z-score*” OR “small for gestational age” OR stunted OR stunting OR starvation OR starved OR starving OR "fetal growth retardation" OR “intrauterine growth restriction” OR IUGR OR "intrauterine growth retardation" OR "fetal growth restriction" OR “bone density” OR “muscle wasting” OR “wasting syndrome” OR “Subjective Global Assessment” OR “alpha tocopherol deficiency” OR “e-hypovitaminosis” OR “e-vitaminosis” OR tocopherol OR “ascorbate deficien*” OR “C-avitaminosis” OR “C-hypovitaminosis” OR “hypo-vitaminosis” OR hypovitaminosis OR “retinol deficien*” OR keratomalacia OR acobalaminosis OR “AdCbl deficien*” OR “adenosylcobalamin deficien*” OR “B12 vitamin*” OR “B 12 vitamin*” OR “Cbl deficien*” OR “Cbl insufficien*” OR “CN-Cbl deficien*” OR “CN-Cbl insufficien*” OR “MeCbl deficien*” OR “MeCbl insufficien*” OR “methylcobalamin deficien*” OR “methylcobalamin insufficien*” OR “calciferol deficien*” OR “calciferol depletion” OR “calciferol insuffien*” OR “colecalciferol deficien*” OR “colecalciferol depletion” OR “colecalciferol insuffien*” OR “D-avitaminosis” OR “menadione deficien*” OR “menadione insufficien*” OR “menadione depletion” OR “menaquinone deficien*” OR “menaquinone insufficien*” OR “menaquinone depletion” OR “phylloquinone deficien*” OR “phylloquinone insufficien*” OR “phylloquinone depletion” OR “cyphytomenadione deficien*” OR “cyphytomenadione insufficien*” OR “cyphytomenadione depletion” OR “corrinoid deficien*” OR “corrinoid insufficien*” OR “corrinoid depletion” OR multivitamin* OR pteroptin OR “tetrahydrofolic acid derivative” OR “muscle atrophy*” OR “osseous density” OR “bone softening” OR “bone decay” OR “bone dysplagia” OR pansteatitis OR “yellow fat disease”)
4	#1 AND #2 AND #3

Abbreviations: *= Truncation and character wildcard symbol; TS= keyword search

F. Cochrane Library Search Strategy

Set #	Search
1	**MeSH descriptor: [Anemia, Sickle Cell] this term only**
2	(sickle cell anemia* OR sickle cell anaemia* OR sickle cell dis* OR sickle cell anemia* OR sickle cell anaemia* OR SCA OR sickle cell disease* OR sickle cell disorder* OR sickling disorder* OR sickler OR hemoglobin S disease* OR haemoglobin S disease* OR HbS disease* OR hemoglobin SC disease* OR haemoglobin SC disease* OR SCD OR HbSC OR Hb SC OR hemoglobin SS disease* OR haemoglobin SS disease* OR HbSS OR Hb SS OR sicle cell OR homozygous sickle OR homozygous SS disease OR homozygous SS sickle cell OR sickle cell hemoglobin C disease* OR sickle cell hemoglobinopath* OR sickle cell haemoglobinopath* OR SC disease* OR sickle cell hemoglobin C OR sickle cell haemoglobin C OR type ss sickle OR bs beta OR sickle OR sickl):ab,ti
3	#1 OR #2
4	**[mh "Pregnant Women"]** OR **[mh Pregnancy]** OR **[mh "Pregnancy Complications"]** OR **[mh "Pregnancy Trimesters"]** OR **[mh Mothers]** OR **[mh "Prenatal Care"]** OR **[mh "Perinatal Care"]** OR **[mh Fetus]** OR **[mh "Infant, Newborn"]** OR **[mh "Maternal Nutritional Physiological Phenomena"]**
5	(pregnan* OR mother* OR fetus* OR newborn* OR neonate* OR preterm infant* OR premature parturition infant* OR premature infant* OR preemie* OR postparturition OR post parturition OR maternal* OR maternity* OR childbearing OR childbirth OR fetomaternal OR fetalmaternal OR foetalmaternal OR postpartum OR post partum OR postpartal OR post partal OR neonatal OR prenatal OR pre natal OR perinatal OR peri natal OR antenatal OR ante natal OR fetal OR foetal OR infant* OR infantile OR baby OR babies OR gestation* OR stillbirth* OR stillborn* OR miscarriage* OR spontaneous abortion* OR obstetric* OR birth* OR born):ab,ti
6	#4 OR #5
7	**[mh "Nutritional Status"]** OR **[mh "Nutritive Value"]** OR **[mh "Nutrition Disorders"]** OR **[mh "Nutrition Assessment"]** OR **[mh Diet]** OR **[mh Malnutrition]** OR **[mh Nutrients]** OR **[mh Micronutrients]** OR **[mh Vitamins]** OR **[mh Provitamins]** OR **[mh " Folic Acid"]** OR **[mh Copper]** OR **[mh "Iron, Dietary"]** OR **[mh Zinc]** OR **[mh Magnesium]** OR **[mh "Recommended Dietary Allowances"]** OR **[mh "Infant Nutrition Disorders"]** OR **[mh "Deficiency Diseases"]** OR **[mh "Vitamin D Deficiency"]** OR **[mh "Vitamin E Deficiency"]** OR **[mh "Vitamin K Deficiency"]** OR **[mh Rickets]** OR **[mh "Rickets, Hypophosphatemic"]** OR **[mh " Familial Hypophosphatemic Rickets"]** OR **[mh "Chronic Kidney Disease-Mineral and Bone Disorder"]** OR **[mh Osteomalacia]** OR **[mh Steatitis]** OR **[mh "Vitamin K Deficiency Bleeding"]** OR **[mh "Birth Weight"]** OR **[mh "Body weigh"]** OR **[mh "Body Mass Index"]** OR **[mh Starvation]** OR **[mh "Bone Density"]**
8	(Nutritional OR nutrition OR diet* OR dietary OR malnutrition OR malnourish* OR undernutrition OR undernourish* OR placental insufficienc* OR alimentary OR food* OR

	nutrient* OR nutritive OR macronutrient* OR micronutrient* OR vitamin* OR provitamin* OR trace element* OR trace mineral* OR Folic acid* OR folate OR ferritin* OR copper OR iron OR zinc OR magnesium OR probiotic* OR symbiotic* OR ascorbic acid OR beta carotene OR biotin OR carnitine OR inositol OR niacin OR pantothenic acid OR pyridoxine OR thiamine OR calcitriol OR cholecalciferol OR ergocalciferols OR iodine OR manganese OR reference daily intake OR recommended daily intake OR recommended daily allowance* OR daily recommended intake OR RDA OR avitaminosis OR avitaminoses OR ascorbic acid deficien* OR scurvy OR scurvies OR hypoascorbemia* OR vitamin C deficien* OR vitamin A deficien* OR vitamin B deficien* OR choline deficien* OR iodine deficien* OR protein deficien* OR hyperhomocysteinemia* OR homocystinuria* OR cystathionine beta synthase deficien* OR CBS deficien* OR pellagra* OR riboflavin deficien* OR thiamine deficien* OR Beriberi OR Wernicke encephalopathy* OR Wernicke* OR Gayet Wernicke* OR vitamin B 12 deficien* OR Addison* anemia* OR subacute combined degeneration* OR subacute combined neuropathy degeneration* OR vitamin B 6 deficien* OR B6 vitamin deficien* OR vitamin B6 deficien* OR pyridoxine deficien* OR vitamin D deficien* OR vitamin E deficien* OR vitamin K deficien* OR rickets OR rachitis OR rachitides OR chronic kidney disease mineral and bone disorder* OR CKD MBD OR renal osteodystroph* OR osteomalacia* OR steatitis OR ascorbic acid insufficien* OR vitamin C insufficien* OR vitamin A insufficien* OR vitamin B insufficien* OR choline insufficien* OR cystathionine beta synthase insufficien* OR CBS insufficien* OR riboflavin insuficien* OR thiamine insufficien* OR vitamin B 12 insufficien* OR vitamin B 6 insufficien* OR B6 vitamin insufficien* OR vitamin B6 insufficien* OR pyridoxine insufficien* OR vitamin D insufficien* OR vitamin E insufficien* OR vitamin K insufficien* OR Biometal* OR vitamin B complex* OR B vitamin* OR neurobion* OR pteroylpolyglutamic acids* OR pteroylpolyglutamates OR tetrahydrofolates OR formyltetrahydrofolates OR formyltetrahydrofolic acids OR leucovorin OR folinic acid OR leukovor* OR citrovorum factor OR folinate OR 5 Formyltetrahydropteroylglutamate OR 5 Formyltetrahydrofolate OR Wellcovorin OR Levoleucovorin OR fusilev OR underweight OR under weight OR birth weight OR birthweight OR body weight OR body mass index OR body mass indice* OR BMI z score* OR small for gestational age OR stunted OR stunting OR starvation OR starved OR starving OR fetal growth retardation OR intrauterine growth restriction OR IUGR OR Intrauterine Growth Retardation OR fetal growth restriction OR bone density OR muscle wasting OR wasting syndrome OR Subjective Global Assessment OR alpha tocopherol deficiency OR e hypovitaminosis OR e vitaminosis OR tocopherol OR ascorbate deficien* OR C avitaminosis OR C hypovitaminosis OR hypo vitaminosis OR hypovitaminosis OR retinol deficien* OR keratomalacia OR acobalaminosis OR AdCbl deficien* OR adenosylcobalamin deficien* OR B12 vitamin* OR B 12 vitamin* OR Cbl deficien* OR Cbl insufficien* OR CN Cbl deficien* OR CN Cbl insufficien* OR MeCbl deficien* OR MeCbl insufficien* OR methylcobalamin deficien* OR methylcobalamin insufficien* OR calciferol deficien* OR calciferol depletion OR calciferol insuffien* OR colecalciferol deficien* OR colecalciferol depletion OR colecalciferol insuffien* OR D avitaminosis OR menadione deficien* OR menadione insufficien* OR menadione depletion OR menaquinone deficien* OR menaquinone insufficien* OR menaquinone depletion OR phylloquinone deficien* OR phylloquinone insufficien* OR phylloquinone depletion OR cyphytomenadione deficien* OR cyphytomenadione insufficien* OR cyphytomenadione depletion OR corrinoid deficien* OR corrinoid insufficien* OR corrinoid depletion OR multivitamin* OR pteroptin OR tetrahydrofolic acid derivative OR muscle atrophy* OR osseous density OR bone softening OR bone decay OR bone dysplagia OR pansteatitis OR yellow fat disease):ab,ti

9	#7 OR #8
10	#3 AND #6 AND #9

Abbreviations: *= Truncation symbol; :ab,ti = Abstract and Title; [mh] = Medical Subject Headings

G. LILACS (Latin America and the Caribbean Literature on Health Sciences) & AIM (African Index Medicus) via WHO Global Index Medicus Search Strategy

Set #	Search
1	**mh:("Anemia, Sickle Cell" OR "Hemoglobin SC Disease")** OR ti:("sickle cell anemia" OR "sickle cell anemias" OR "sickle cell anaemia" OR "sickle cell anaemias" OR sca OR "sickle cell disease" OR "sickle cell diseases" OR "HbS disease" OR "hemoglobin SC disease" OR "hemoglobin SC diseases" OR "haemoglobin SC disease" OR hbsc OR "Hb SC" OR "hemoglobin SS disease" OR "hemoglobin SS diseases" OR "haemoglobin SS disease" OR "haemoglobin SS diseases" OR hbss OR "Hb SS" OR "sicle cell" OR scd/sca OR sca/scd OR "homozygous sickle" OR "homozygous SS disease" OR "homozygous SS sickle cell" OR "sickle cell hemoglobin C disease" OR "sickle cell hemoglobin C diseases" OR "SC disease" OR "SC diseases" OR "sickle cell hemoglobin C" OR "sickle cell haemoglobin C" OR “type ss sickle” OR “hbs beta” OR sickl) OR ab: ("sickle cell anemia" OR "sickle cell anemias" OR "sickle cell anaemia" OR "sickle cell anaemias" OR sca OR "sickle cell disease" OR "sickle cell diseases" OR "HbS disease" OR "hemoglobin SC disease" OR "hemoglobin SC diseases" OR "haemoglobin SC disease" OR hbsc OR "Hb SC" OR "hemoglobin SS disease" OR "hemoglobin SS diseases" OR "haemoglobin SS disease" OR "haemoglobin SS diseases" OR hbss OR "Hb SS" OR "sicle cell" OR scd/sca OR sca/scd OR "homozygous sickle" OR "homozygous SS disease" OR "homozygous SS sickle cell" OR "sickle cell hemoglobin C disease" OR "sickle cell hemoglobin C diseases" OR "SC disease" OR "SC diseases" OR "sickle cell hemoglobin C" OR "sickle cell haemoglobin C" OR “type ss sickle” OR “hbs beta” OR sickl)
2	**mh:("Pregnant Women" OR g08.686.784.769* OR c12.050.703* OR g08.686.707* OR Mothers OR ”Prenatal Care” OR e02.760.703* OR ”Perinatal Care” OR a16.378* OR m01.060.703.520* OR g07.203.650.566*)** OR ti:(mother* OR pregnan* OR fetus* OR newborn* OR neonate* OR maternal* OR maternity OR childbearing OR childbirth OR postpartum OR “post partum” OR postpartal OR “post partal” OR neonatal OR prenatal OR “pre natal” OR perinatal OR “peri natal” OR antenatal OR “ante natal” OR fetal OR foetal OR infant* OR infantile OR baby OR babies OR gestation* OR stillbirth* OR stillborn* OR miscarriage* OR “spontaneous abortion” OR “spontaneous abortions” OR obstetric* OR birth* OR born) OR ab:(mother* OR pregnan* OR fetus* OR newborn* OR neonate* OR maternal* OR maternity OR childbearing OR childbirth OR fetomaternal OR fetalmaternal OR foetalmaternal OR postpartum OR “post partum” OR postpartal OR “post partal” OR neonatal OR prenatal OR “pre natal” OR perinatal OR “peri natal” OR antenatal OR “ante natal” OR fetal OR foetal OR infant* OR infantile OR baby OR babies OR gestation* OR stillbirth* OR stillborn* OR miscarriage* OR “spontaneous abortion” OR “spontaneous abortions” OR obstetric* OR birth* OR born)
3	**mh:(”nutritional status” OR ”nutritive value” OR ”nutrition disorders” OR ”nutrition assessment” OR diet OR “dietary supplements” OR nutrients OR ”trace elements” OR "recommended dietary allowances")** OR **mh:(c18.654* OR g07.203.650.240* OR g07.203.300.456* OR d27.505.696.494* OR c18.654.521* OR g07.203.300.681*)** OR ti:(nutritional OR nutrition OR nutrient* OR nutritive OR macronutrient* OR micronutrient* OR vitamin* OR provitamin* OR diet* OR dietary OR malnutrition OR malnourish* OR

	undernutrition OR undernourish* OR “placental insufficiency” OR “placental insufficiencies” OR alimentary OR food* OR nutrient* OR nutritive OR macronutrient* OR vitamin* OR provitamin* OR "trace element" OR “trace elements” OR “trace mineral” OR “trace minerals” OR "reference daily intake" OR "recommended daily intake" OR "recommended daily allowance" OR "recommended daily allowances" OR "daily recommended intake" OR rda) OR ab:(nutritional OR nutrition OR nutrient* OR nutritive OR macronutrient* OR micronutrient* OR vitamin* OR provitamin* OR diet* OR dietary OR malnutrition OR malnourish* OR undernutrition OR undernourish* OR “placental insufficiency” OR “placental insufficiencies” OR alimentary OR food* OR nutrient* OR nutritive OR macronutrient* OR vitamin* OR provitamin* OR "trace element" OR “trace elements” OR “trace mineral” OR “trace minerals” OR "reference daily intake" OR "recommended daily intake" OR "recommended daily allowance" OR "recommended daily allowances" OR "daily recommended intake" OR rda)
4	#1 AND #2 AND #3

Abbreviations: * = Truncation symbol; mh = Global Index Medicus descriptor; ti = Title

Notes:

g08.686.784.769* = Pregnancy [mh:explode]
c12.050.703*= Pregnancy Complications [mh: explode]
g08.686.707* = Pregnancy Trimesters [mh:explode]
E02.760.703* =Perinatal Care [mh:explode]
a16.378*= Fetus [mh:explode]
m01.060.703.520*= Infant, Newborn [mh:explode]
g07.203.650.566* = Maternal Nutritional Physiological Phenomena [mh:explode]
g07.203.650.660* = Nutritive Value [mh:explode]
c18.654* = Nutrition Disorders [mh:explode]
g07.203.650.240* = Diet [mh:explode]
g07.203.300.456* = Dietary Supplements [mh:explode]
g07.203,300,681* = Nutrients[mh:explode]
c23.888.144* = Body Weight[mh:explode]
